# Complete Whole-Genome Sequences of Two Raoultella terrigena Strains, NCTC 13097 and NCTC 13098, Isolated from Human Cases

**DOI:** 10.1128/MRA.00239-19

**Published:** 2019-07-03

**Authors:** Mohammed-Abbas Fazal, Sarah Alexander, Nicholas E. Grayson, Ana Deheer-Graham, Karen Oliver, Nancy Holroyd, Julian Parkhill, Julie E. Russell

**Affiliations:** a Culture Collections, Public Health England, London, United Kingdom; b Wellcome Trust Genome Campus, Wellcome Trust Sanger Institute, Hinxton, United Kingdom; University of Arizona

## Abstract

Raoultella terrigena is a bacterial species associated with soil and aquatic environments; however, sporadic cases of opportunistic disease in humans have been reported. Here, we report the first two complete genome sequences from clinical strains isolated from human sources that have been deposited in the National Collection of Type Cultures (NCTC).

## ANNOUNCEMENT

Raoultella terrigena (previously known as Klebsiella terrigena) is a Gram-negative rod-shaped nonmotile bacterium first isolated in 1981 (1). Raoultella terrigena is generally considered to be an environmental bacterium and is isolated mainly from environmental sites such as soil and aquatic environments (2); however, this species has also been described as being involved in opportunistic human infections (3, 4). To date, four draft genomes are available for this bacterial species from strains isolated from the environment (soil and nematode sources) (5). Here, we report the first complete whole-genome sequences isolated from human infections for Raoultella terrigena strains NCTC 13097 and NCTC 13098. These strains were deposited in the NCTC and were isolated in 1990 from the tonsils of a child (NCTC 13097) and sputum of a patient with chronic bronchitis (NCTC 13098) (6).

The strains were recovered from lyophilized ampules on nutrient agar and incubated at 37°C for 24 h. Genomic DNA was extracted from the resulting bacterial lysate using the MasterPure DNA kit and underwent quality controls for high-molecular-weight DNA (>60 kbp) using the Agilent 2200 TapeStation and high yield using a Qubit fluorometer (minimum 3 μg DNA). Sequencing was performed on the Pacific Biosciences (PacBio) RS II platform. A 10- to 20-kb library was prepared and sequenced using C4-P6 chemistry on single-molecule real-time (SMRT) cells, with a 180-min collection protocol, on the PacBio RS II platform. Sequence reads were assembled using HGAP v3 (7) of the SMRT Analysis software v2.3.0 (PacBio). The fold coverage to target when picking the minimum fragment length for assembly was set to 30, and the approximate genome size was set to 3 Mbp. The assembly was circularized using Circlator v1.1.3 (8). Finally, the circularized assembly was polished using the PacBio RS_Resequencing protocol and Quiver v1 of the SMRT Analysis software v2.3.0 (PacBio). Automated annotation was performed using Prokka v1.5 (9) and genus-specific databases from RefSeq (10).

The chromosome of NCTC 13097 was assembled into two contigs of 5,574,669 bp, with a GC content of 57.3% and an *N*_50_ value of 177,116 bp. The average read coverage for the assembly was 118×. There were 5,386 protein-coding (CDS) genes, 84 tRNA genes, and 25 rRNA genes. The strain contained one plasmid, which was assembled into a single contig of 86,732 bp, with an average read coverage for the assembly of 116× and a GC content of 50.9%, The presence of a plasmid contig was further confirmed using the Web-based mlplasmid Shiny app v1.0.0, with a probability score of 0.985 (11).

The chromosome of NCTC 13098 was assembled as a single contig of 6,073,951 bp, with a GC content of 57%. The average read coverage for the assembly was 40×, with an *N*_50_ value of 56,385 bp. The strain did not contain any plasmids. There were 5,796 protein-coding (CDS) genes, 85 tRNA genes, and 25 rRNA genes. In both strains, the fosfomycin resistance gene *fosA* was identified using ResFinder version 2.1 (12).

### Data availability.

The complete genome sequences have been deposited in the European Nucleotide Archive under accession numbers SAMEA3368263 and SAMEA3368264 for NCTC 13097 and NCTC 13098, respectively, within study accession number PRJEB6403. The Sequencing Read Archive data are located under accession numbers ERX1057486 and ERX1045254 for NCTC 13097 and NCTC 13098, respectively.
